# Multiverse analysis and the Bradley-Terry model: A proposed approach for evaluating palatability and preference

**DOI:** 10.3168/jdsc.2023-0500

**Published:** 2024-03-02

**Authors:** A.E. Pape, R.A. Scuderi, J. Green, C.S. Ballard

**Affiliations:** 1 William H. Miner Agricultural Institute, Chazy, NY 12921; 2 Lallemand Specialties Inc., Milwaukee, WI 53218

## Abstract

The palatability of feed for dairy cows is an important consideration but is difficult to measure, particularly when considering more than 2 feeds. We outline how a combination of multiverse analysis and Bradley-Terry modeling, 2 methodological tools that have rarely been applied in dairy science, can be adapted to address this problem. Specifically, we propose to apply multiverse analysis as a way to consider a range of thresholds for how much of a mixed grass-legume (MGL) silage had to be consumed (as a percent of the total DMI) to be designated as preferred. Each threshold gives rise to a separate dataset and a corresponding fitted Bradley-Terry model. Bradley-Terry models attribute to each feed what is commonly referred to as an “ability” in the context of sports or other competitions but can be interpreted as palatability when applied to feeds. This combined approach is a way of estimating palatabilities that appropriately reflect the degree of preference cows express through their feeding behavior. It has the advantages of being transparent and relatively easy to implement. A possible disadvantage is that this method is limited to a paired comparison approach and has difficulties with main-effects statistical inference. We demonstrate the use of this methodology on an example dataset comparing MGL silages under different ensiling conditions and exposed to oxygen for different durations.

## Highlights

• We propose using multiverse analysis and Bradley-Terry modeling.
• These methods are useful for determining direction and degree of preference.
• This can apply to any paired comparisons study of palatability.

## Graphical Abstract

**Figure:**
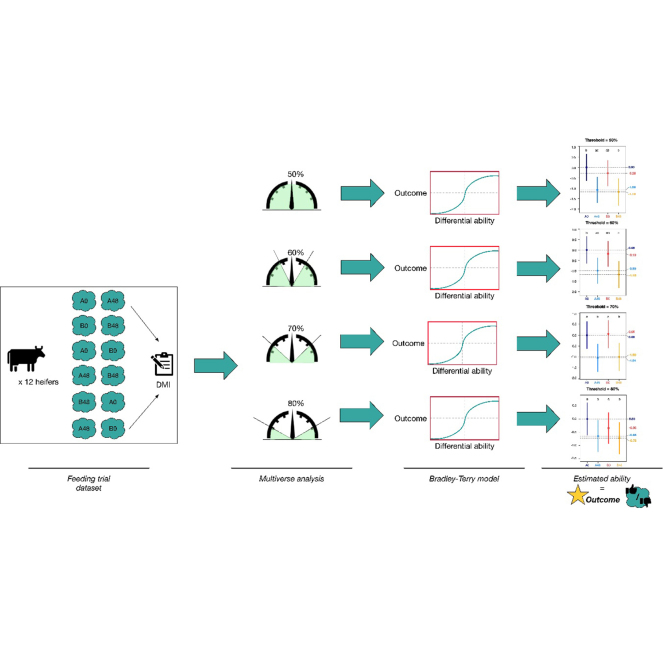
**Summary** Grass silage from 2 different ensiling conditions (bunkers A and B) was offered fresh (0) or after 48 hours of oxygen exposure (48) to a group of 12 pregnant Holstein heifers (mean ± standard deviation; body weight 525 ± 49 kg, days carried calf 44 ± 5). Animals were offered 2 of the 4 different treatments in different combinations across the preference trial (6 days), and dry matter intake was measured after 30 minutes. Four separate versions of the dry matter intake dataset were then created: 1 for each threshold of what percentage of intake had to come from a particular feed for it to count as preferred (this processing stage can be considered a form of multiverse analysis). The thresholds were 50%, 60%, 70%, and 80%. A Bradley-Terry model was then fitted to each dataset, and its output was used as an estimate of palatability. This combination of analysis techniques is a means of determining what feed types cows prefer and how strongly they prefer them.

Feed quality is crucial to intensive dairy production systems. One important aspect of feed quality that is difficult to characterize is palatability. Palatability is often characterized on the basis of chemical, microbial, or organoleptic factors upon which palatability is known to depend (Kung et al., 2018). An alternative approach is to estimate palatability from cows' feeding behavior, specifically based on their greater or less intake of one feed rather than another. These approaches are often used in tandem, with some background information on chemical, microbial, or organoleptic factors brought to bear on behavioral data (e.g., Spörndly and Åsberg, 2006; Miller-Cushon et al., 2014). While an animal's intake has been regarded as an accurate indicator for palatability, using it as a single factor relies on several assumptions regarding the management and well-being of animals enrolled in a study. Dry matter intake remains a good proxy for determining preference when offered 2 or more feeds under controlled conditions. Gerlach et al. (2014) examined the impact of changes in organoleptic aspects in grass silage and reported decreases in DMI when grass silage was aerobically exposed by applying multi-dimensional scaling (**MDS**) to differences in consumption of each pair of simultaneously offered feeds. Multi-dimensional scaling refers to a class of algorithms designed to configure a set of points in a space such that the distances among them are approximately the same as a set of distances specified by the user. The distances need not be literal geometric distances. For instance, dissimilarity is analogous to distance, so estimates of dissimilarity can be used instead, provided the estimation is made in a reproducible manner. (Several accessible overviews of MDS are available; for instance, Hout et al., 2013.) However, preference is not necessarily analogous to distance because it has both a magnitude and a direction (or valence), whereas distance has only magnitude (i.e., there cannot be negative distances). Multi-dimensional scaling is thus inappropriate for preferences. Carroll et al. (2023) used sequential elimination, where all feed types were made available to the cows for a period of time, the most-consumed feed (highest DMI) was removed, and the process was repeated until only one feed type remained. This yielded a ranking of all feed types, with the order in which they were removed being the decreasing order of preference. Treatment rankings were analyzed using a Plackett-Luce model. A disadvantage of this method is that it is not able to account for the degree of feed preference (i.e., how much more of the preferred feed was consumed). Furthermore, there are a limited number of studies on mature dairy cows using DMI as a proxy for palatability, offering few recommendations for analysis. Considering the growing importance of palatability studies for the dairy industry, the implementation of a statistical methodology is important to help make informed and accurate conclusions.

As an alternative to MDS and the use of the Plackett-Luce model previously reported, the present work demonstrates a methodology consisting of multiverse analysis followed by Bradley-Terry modeling and applies it to an example dataset. Multiverse analysis (Steegen et al., 2016) is an increasingly popular strategy for drawing empirical conclusions that are robust to arbitrary decisions made during data processing. In short, multiverse analysis consists of testing a hypothesis on multiple versions of a dataset and considering the results collectively. Ordinarily, data processing decisions that are left to the discretion of the investigator, such as cutpoints for continuous variables and data exclusion, can have an unwarranted influence on the conclusions of the analysis. Rather than advocating any one alternative, multiverse analysis prescribes creating a version of the data for every reasonable alternative and applying the same model fitting process to all. Instead of a single hypothesis test and associated *P*-value, which is often misleadingly definitive, the output of multiverse analysis is an ensemble of tests. Ideally, a positive or negative result will predominate within this ensemble, thereby confirming that no one processing decision had undue influence. Even if no result predominates, multiverse analysis will have prevented a conclusion from being drawn prematurely.

Bradley-Terry modeling is an approach to paired comparisons that has been widely used in analysis of both animal and human behavior, although to the best of our knowledge it has rarely been applied to feed preference studies for dairy cows. A Bradley-Terry model attributes an “ability” to the entities that are competition with one another (e.g., sports teams). The probability that any given team (to use the sports analogy) will defeat another given team is a function of the difference of their respective abilities. The structure of a Bradley-Terry model is analogous to a logistic regression in which the game is the unit of observation, the outcome is the response variable, and the respective abilities are the predictors (or, more precisely, the predictors are the identities of the teams involved and the abilities are their coefficients). Unlike an ordinary logistic regression, a Bradley-Terry model can take on a mixed-effects structure, although use of such capability was beyond the scope of the present demonstration. Turner and Firth (2012) published an accessible introduction to Bradley-Terry in conjunction with its implementation in R. As a term, “ability” has connotations of action and autonomy that cease to be appropriate when taken out of a sports context and applied to animal feed. As a result, when necessary, we substitute the term “palatability” for the sake of clarity; however, the underlying mathematics remain unchanged. Moreover, like “ability,” “palatability” is here meant as a tool for quantifying a particular behavior while bracketing questions about what exactly the palatability consists of in qualitative terms for the animal (e.g., the sensory input the animal receives from the feed, and the process of selecting one over the other). In reporting a given feed's palatability as a scalar quantity, we are simply saying that the cows behaved as if that feed had that palatability. To return to the sports analogy, to say that a team has a certain Bradley-Terry ability is simply to say that its past performance (and likely future performance) is well described by that estimate; the qualitative complexity of what ability consists of in any given sport or athletic competition is a separate matter.

The present study proposes an alternative analysis approach that consists of combining methods from multiverse analysis and the Bradley-Terry model, focusing on the use of behavioral data rather than chemical, microbial, or organoleptic data. Using a dataset from a previous trial as an example, the null hypothesis is that all feed types are equally palatable (alternatively, that some are more palatable than others). We measured preference using DMI from the example dataset as a proxy when offering mixed grass-legume (**MGL**) silage to pregnant Holstein heifers from 2 bunkers (bunker **A** and bunker **B**) immediately after defacing (0) or after 48 h of aerobic exposure (48). It should be noted that we are employing this dataset as a testbed for the proposed methodology; our aim is not to draw conclusions about the effects of these particular ensiling conditions or of aerobic exposure time. Moreover, our aim was not to directly compare this method or develop a ranking of methods, but rather to demonstrate this method and assess some of its advantages and disadvantages.

The dataset originated from a study that was conducted at the William H. Miner Agricultural Research Institute (Chazy, NY) from July 24, 2021, through August 4, 2021. All experimental procedures involving pregnant heifers were approved by the William H. Miner Agricultural Research Institute Animal Care and Use Committee (IACUC #2021AUR07). A 16 ha field of grass/alfalfa (predominantly grass) was ensiled (MGL) under 2 different ensiling conditions (bunker A and bunker B). Twelve pregnant Holstein heifers (16–21 mo of age; mean ± SD; BW 525 ± 49 kg, days carried calf 44 ± 5) were enrolled and housed in tiestalls where they were allowed to adapt for 7 d before first study feeding. Heifers were fed TMR formulated to meet their requirements according to Cornell Net Carbohydrate and Protein System version 6.5 (Cornell University, Ithaca, NY; 69.5 MGL silage, 20.3 corn silage, 10.2 concentrate; %, DM basis) ad libitum and each stall was equipped with a water bowl. The TMR was removed 1 h before MGL silage being offered. Initially, 90 kg of each MGL silage (bunker A and bunker B) was removed from the bunks daily and placed into forage carts. Forage carts were stored out of direct sunlight in an open area at ambient temperature. Heifers were introduced to 1 of 4 treatments for 3 h a day for the first 4 d representing silo and aerobic exposure: A freshly defaced (**A0**), A 48 h after defacing (**A48**), B freshly defaced (**B0**), and B 48 h after defacing (**B48**). This adaptation period allowed each heifer to experience all 4 treatments individually over the 4 d period and associate the MGL silage with postingestive metabolic response, taste and smell produced by the MGL silage (Gerlach et al., 2014). After 3 h, the MGL silages were removed and a premixed TMR was fed ad libitum for the remainder of the day. Illness would be the main reason to exclude an animal from a study of this kind; no illness or adverse health events were reported during the study.

During the treatment period, palatability of forage treatments was evaluated by offering each heifer 2 bins (30.5 cm × 35.5 cm × 58.4 cm each) of different forage treatments in the feedbunk area for a total time of 3 h for 6 consecutive days. With 4 feed types, there were 6 combinations; each heifer received each combination exactly once over the course of the 6 d of the treatment period. The left/right orientation in which the combinations were offered to the heifer was randomized. Treatment intakes were measured after 30 min and 3 h. For the remainder of the day, heifers were fed a premixed TMR ad libitum for 10% refusals and with mean ± SD DMI of 8.3 ± 0.8 kg/d. All forage treatments and TMR were sampled daily and dried in a forced-air oven at 105°C for 18 to 24 h for DM calculation. Following Gerlach et al. (2014), DMI was calculated for each heifer for 30 min and 3 h MGL silage and daily TMR. Animals were offered a mean of 2.64 kg of MGL silage on a DM basis in each bin (2.09, 2.99, and 0.22 kg; minimum, maximum, and SD, respectively). Of the MGL silage, animals consumed an average of 0.48 kg (0.0, 1.68, and 0.40 kg; minimum, maximum, and SD, respectively) after 30 min and an average of 0.98 kg (0.0, 4.09, and 0.82 kg; minimum, maximum, and SD, respectively) after 3 h. Both 30 min intake and 3 h intake follow a strongly asymmetric distribution, so the latter has a higher SD than the former simply by having a higher mean. The 30 min and 3 h intakes were found to be highly correlated (r ≈ 0.89), which demonstrates a shorter feeding duration is suitable for measuring preference in this study design. Hence, for the purpose of demonstrating the application of a 2-step analysis approach, only 30 min intakes are used.

Data were analyzed in R version 4.2.2 (R Core Team, 2022). We applied multiverse analysis (in a limited form) to the choice of a threshold for DMI. In using DMI as an indicator for preference, we assumed optimal health, dietary, and management conditions were met. We experimented with thresholds of 50%, 60%, 70%, and 80% as criteria for how much of a MGL silage had to be consumed (as a percent of the total intake after 30 min) to be designated as preferred. These thresholds are referred to hereafter as “win-loss-tie” thresholds. Any cases in which neither feed reached the threshold were retained in the model as ties. The percentage of cases that were retained as ties were 0%, 14%, 32%, and 54% for thresholds of 50%, 60%, 70%, and 80%, respectively. We implemented the Bradley-Terry model through the BradleyTerry2 package (Turner and Firth, 2012). Statistical inference for differences between Bradley-Terry abilities was conducted using a pooled SE calculated from quasi-SE of each individual palatability as described in Firth and de Menezes (2004). The multiverse analysis was written in R (data and R code are available in the Dryad repository; see Notes). Thus, a R package was not required for this framework.

By using a multiverse analysis followed by the Bradley-Terry model, we are proposing an approach that can allow for accurate estimation of both degree and direction of feed preference. The behavior recorded in the sequential elimination procedure described by Carroll et al. (2023) consisted of an ordering of the feed types being studied. The margin by which each feed type was preferred at each stage of the elimination was not recorded (or at least not used). With the intent of ameliorating this kind of limitation, we applied a version of multiverse analysis to a range of degrees of preference. It should be noted this limitation is not inherent to either sequential elimination or Plackett-Luce modeling; a multiverse analysis is, in principle, compatible with both.

Figure 1 shows palatabilities as estimated by the Bradley-Terry method for 4 selected win-loss-tie thresholds along with 95% CI based on quasi-SE. Mixed grass-legume silage corresponding to A0 was designated as the reference level and so was assigned a palatability of zero. Because all other palatabilities are measured relative to A0 and heifers often preferred A0, other palatabilities frequently take on negative values. This does not imply that they are unpalatable in absolute terms; their palatability is negative only as a result of being measured on this particular scale. The *P*-values corresponding to significances indicated in Figure 1 are shown in Table 1. Both Figure 1 and Table 1 support approximately the same set of conclusions. Specifically, there is a difference between MGL silages with different exposure times but not between MGL silages from different bunks.

**Figure 1:**
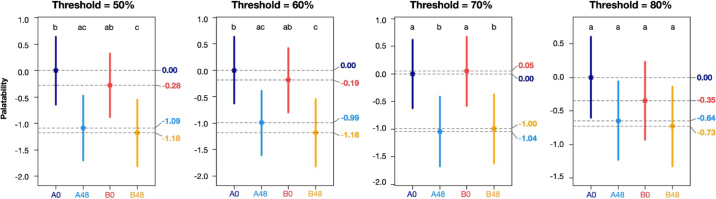
Estimates and 95% CI of palatability by treatment for selected win-loss-tie thresholds applied to 30-min intake data (palatabilities with different letters differ significantly, *P* ≤ 0.05).

**Table 1:** *P*-values for tests of palatability differences between treatments based on quasi-SE for selected win-loss-tie thresholds applied to 30-min intake data

Item	Threshold = 50%			Threshold = 60%			Threshold = 70%			Threshold = 80%		
	A0	A48	B0	A0	A48	B0	A0	A48	B0	A0	A48	B0
B48	0.009	0.83	0.04	0.009	0.67	0.02	0.02	0.92	0.02	0.09	0.84	0.35
B0	0.53	0.06	—	0.67	0.06	—	0.92	0.01	—	0.41	0.47	—
A48	0.015	—	—	0.02	—	—	0.02	—	—	0.13	—	—

Multiverse analysis entails use of an ensemble of datasets, each processed in a slightly different way. This ensemble will collectively yield either redundant or contradictory results. From one point of view, this ensemble may seem to be an unnecessary complication if the results are redundant and an impediment to clarity if they are conflicting. In contrast, the intent of multiverse analysis is to embrace the redundancy or contradiction as a source of evidence. Redundant results are stronger than any one version of the data on its own. Conflicting results suggest that the results of any one version of the data in isolation are tenuous. Using the DMI dataset described above, multiverse analysis yielded redundant results with most thresholds suggesting similar conclusions. However, the multiverse analysis results we obtained were not completely redundant. In particular, at an 80% threshold, although the estimated palatabilities were higher for 0 h than for 48 h MGL silages, the differences were no longer significant. This illustrates a strength of multiverse analysis: by considering the results of all thresholds jointly, we avoid being misled by any one individual threshold's results viewed in isolation. A study that used only the 80% threshold might underestimate the degree of preference. The results corresponding to each threshold provide context for the others; no one threshold is definitive.

We chose to apply multiverse analysis to one particular aspect of data processing: the win-loss-tie threshold. It is worth considering in more detail what role the win-loss-tie threshold plays in our interpretation of the heifers' behavior. At one extreme, the preferred feed could be simply determined based upon the heifer's consumption, regardless of how much more was consumed. This definition makes no distinction between mild and strong preference as it does not consider “margin of victory.” At the opposite extreme, a feed could be defined as preferred only if at least 80% of the heifer's intake was from that feed. If neither feed reached the 80% threshold, the heifer would be considered indifferent between feeds. This definition avoids conflating mild and strong preferences, but at the cost of simply ignoring mild preferences. Multiverse analysis allows us to make the most of the information the heifer conveys through intake by not reducing it to a single decisive verdict in favor of one feed or another. Finally, expressing palatability in terms of Bradley-Terry abilities is a way of preserving the degree of preference in the final results.

A limitation of the proposed method is it applies only to feed preference studies that take a paired comparisons approach for both data collection and hypothesis testing. Note that the former does not entail the latter; some studies may collect data in a paired comparisons format but do so with the aim of testing the effect of some feature of the feed types (e.g., concentration of a specific ingredient) on palatability rather than testing the difference in palatability between any specific pair of feed types. At least in present form, the proposed method's null hypothesis is that all feed types are equally palatable (alternatively, that some are more palatable than others). In other words, we apply a separate hypothesis test to each pair of MGL silages in isolation. Any hypothesis test involving 3 or more palatabilities (i.e., the effect of some predictor on ability) would require a different testing format. For instance, our testing format could not accommodate a test of the main effect of MGL silage type (i.e., whether the A MGL silages differed from the B MGL silages), or a test of the main effect of exposure time (i.e., whether 0 h differed from 48 h), or a test of the interaction of type and time. In principle, the Bradley-Terry method can accommodate main effects and interactions, but that is an extension of the method beyond the scope of the present work.

An additional challenge we experienced when using this dataset to demonstrate the proposed approach was power. We estimated power for a unit difference between the two 48-h palatabilities (i.e., for an effect configuration consisting of all palatabilities equal to zero except for one of the 48-h palatabilities, which was set to −1). The Bradley-Terry method assumes that if 2 feeds differ in palatability by 1 unit, the probability that a cow will select the more palatable feed is 1/(1 + *e*^−1^) = 73% (Turner and Firth, 2012). For such an effect, the power was approximately 64% with the current experimental setup (6 pairs offered once to 12 heifers for a total of 72 offerings). To achieve higher power, more heifers would be needed or they would need to be offered each pair of feeds more than once. An alternative means of improving power would be to reduce the number of feeds. With 3 MGL silages instead of 4, assuming that 72 offerings were still observed, power increases to approximately 82%. It is important to note that power applies to each branch of the multiverse (i.e., each threshold) individually, but not to the multiverse as a whole. Power is the property of a formal statistical test in conjunction with a sample size and data distribution; the multiverse approach as a whole does not constitute a single formal test but rather an ensemble of tests (or, more precisely, an ensemble of datasets, each subjected to a separate test).

Furthermore, it is important to note this technique was applied to a dataset that originated from a short-term study which used methods from Gerlach et al. (2014). Those authors implemented a short-term duration for their preference studies with adaptation and experimental periods to match the number of different feed combinations in a pairwise manner. This design was based on previous studies performed in pigs and goats (Kyriazakis et al., 1990; Burns et al., 2001). Palatability will change over time due to acclimation, variations in the feed itself, as well as other factors. Therefore, the approach presented in our work remains suitable for short-term studies that minimize the potential for acclimation to all feed types as a result of prolonged offerings.

The approach presented here, consisting of the use of a multiverse analysis followed by the Bradley-Terry model, is a way of estimating palatabilities that appropriately reflect the degree of preference cows express through their feeding behavior. Using data from a previous experiment as an example, the new approach was able to detect differences between periods of oxygen exposure (0 and 48 h). However, it was not able to distinguish any differences between the different bunkers (A and B). This approach has the advantages of being transparent and relatively easy to implement. A possible disadvantage is that this method is limited to a paired comparison approach and has difficulties with main-effects statistical inference. Future trials evaluating palatability of feeds using pairwise comparisons in a short-term study can use this proposed methodology to determine the degree and direction of preference for a particular feed.
